# Mammographic density and its interaction with other breast cancer risk factors in an Asian population

**DOI:** 10.1038/sj.bjc.6606085

**Published:** 2011-01-18

**Authors:** C S Wong, G H Lim, F Gao, R W Jakes, J Offman, K S Chia, S W Duffy

**Affiliations:** 1 Centre For Molecular Epidemiology, National University of Singapore, Blk MD3, 16 Medical Drive, Singapore 117597, Singapore; 2 Investigational Medicine Unit, National University Health System, National University Hospital, 5 Lower Kent Ridge Road, Kent Ridge Wing 2, Level 6, Singapore 119074, Singapore; 3 Division of Clinical Trials and Epidemiological Sciences, National Cancer Centre, 11 Hospital Drive, Singapore 169610, Singapore; 4 Health Services and Systems Research, Duke-NUS Graduate Medical School, 8 College Road, Singapore 169857, Singapore; 5 WorldWide Epidemiology, GlaxoSmithKline, 150 Beach Road, #22-00 Gateway West, Singapore 189720, Singapore; 6 Cancer Prevention Trials Unit, Cancer Research UK Centre for Epidemiology, Mathematics and Statistics, Wolfson Institute of Preventive Medicine, Barts and The London School of Medicine and Dentistry, Queen Mary, University of London, Charterhouse Square, London EC1M 6BQ, UK; 7 Department of Epidemiology and Public Health, Yong Loo Lin School of Medicine, National University of Singapore, Blk MD3, 16 Medical Drive, Singapore 117597, Singapore; 8 Wolfson Institute of Preventive Medicine, Barts and The London School of Medicine and Dentistry, Queen Mary, University of London, Charterhouse Square, London EC1M 6BQ, UK

**Keywords:** Asian population, body mass index, breast cancer, breast density, mammography

## Abstract

**Background:**

Joint effects of mammographic density and other risk factors on breast cancer risk remain unclear.

**Methods:**

From The Singapore Breast Screening Project, we selected 491 cases and 982 controls. Mammographic density was measured quantitatively. Data analysis was by conditional logistic regression.

**Results:**

Density was a significant risk factor, adjusting for other factors. Density of 76–100% had an odds ratio of 5.54 (95% CI 2.38–12.90) compared with 0–10%. Density had significant interactions with body mass index and oral contraceptive use (*P*=0.02).

**Conclusions:**

Percent density increases breast cancer risk in addition to effects of other risk factors, and modifies the effects of BMI and OCs.

Radiologically dense breast tissue is associated with an increased risk of breast cancer (Sala *et al*, 1998; McCormack and dos Santos Silva, 2006; Boyd *et al*, 2007). Breast density is also positively correlated with some breast cancer risk factors and negatively correlated with others, notably body mass index (BMI) and age (Boyd *et al*, 1998; Jakes *et al*, 2000; Sala *et al*, 2000). Unresolved issues include the extent to which density contributes to risk independently of other risk factors, how it interacts with other risk factors (that is, whether the effect of density is modified in the presence of other factors), whether absolute dense area or percent density is a better risk predictor and the possible interplay between ethnic origin and density (Maskarinec *et al*, 2002; Duffy *et al*, 2004; Chen *et al*, 2006).

We previously reported on a case-control study of 174 breast cancer cases and 348 controls nested within the Singapore Breast Screening Project (SBSP) using the Tabar qualitative measure of breast density (Jakes *et al*, 2000; Duffy *et al*, 2004). The major finding of this study was a significant increase in risk with the Tabar mammographic pattern IV (Jakes *et al*, 2000). We have now enhanced the same study to include 491 cases and 982 controls, and using a quantitative measure of density, we address the following issues:

1. The association of density with other breast cancer risk factors;
2. The additional effect of density on risk, after adjusting for other risk factors;
3. Interactions between breast density and other risk factors; and
4. Differences between Chinese and other ethnic groups with respect to density and its effect on risk.

## Materials and methods

As part of the SBSP, 29 193 Singaporean women aged 45–69 years were screened with a single two-view mammography between 1994 and 1997 (Ng *et al*, 1998). All subjects completed a questionnaire covering the major breast cancer risk factors (Jakes *et al*, 2000). Breast cancer cases were notified as either detected through the SBSP or subsequently through record linkage with the Singapore Cancer Registry. This study was approved by the National University of Singapore Institutional Review Board. For each cancer case, we selected two controls, who had not been diagnosed with breast cancer, matched for age, ethnicity and time of screening. The original mammograms from cancer patients and controls were retrieved and density was measured using the Cumulus interactive threshold method (Byng *et al*, 1998). This estimates both total breast area, total area of dense tissue and percent density, calculated as the quotient of the total area of dense tissue and the total breast area, multiplied by 100. We analysed a total of 491 breast cancer cases (122 diagnosed at screening and 369 subsequently – we were unable to retrieve the mammograms for 10 cases in the previous study) and 982 controls with density measures.

Associations of mammographic density with other breast cancer risk factors among controls only were assessed using the *χ*^2^-test. The effects of density breast cancer risk was assessed using conditional logistic regression (Breslow and Day, 1980). Interactions of density with other risk factors in their joint effects on risk were also evaluated using conditional logistic regression.

## Results

Table 1 shows the distributions of breast density and other breast cancer risk factors for cases and controls. In univariate conditional logistic regression, significantly increased risks were observed for higher percent density (*P*<0.001), higher BMI (*P*=0.03), low parity (*P*=0.005), early menarche (*P*=0.003), family history of breast cancer (*P*<0.001), high socioeconomic status as measured by education (*P*=0.001), later age at menopause (*P*=0.008) and ever use of hormone replacement therapy (HRT) (*P*=0.008). Significantly reduced risks were associated with breast feeding (*P*=0.006) and early age at first birth (*P*=0.01). In addition, a significantly increased risk (*P*=0.001) was observed with higher total dense area and a significantly reduced risk (*P*=0.002) with higher total breast area (data not shown). Percent breast density had a stronger effect on risk than total dense area or total breast area. We therefore used percent density in subsequent analyses.

After entering the nonmammographic variables into stepwise conditional logistic regression, breast feeding and socioeconomic status were no longer significant. The remaining factors were used for multivariable adjustment of the effect of density. Table 2 shows the odds ratios (OR) associated with density, unadjusted, adjusted only for BMI and adjusted for all significant risk factors. Density had a stronger effect after adjustment for BMI, consistent with the negative confounding effect. Density remained a significant risk factor after adjustment for other risk factors (*P*<0.001). After multivariable adjustment, the 76–100% density category had an OR of 5.54 (95% CI 2.38–12.90) compared with 0–10%.

We estimated the effect of percent density on risk of cancers by time since screen (detailed results available from the authors). The trend remained highly significant 6 or more years after the mammogram, although its magnitude showed a tendency to decrease with time.

We also tabulated percent density against various breast cancer risk factors within the controls (data available from the authors). Density was significantly increased in association with low BMI (*P*<0.001), nulliparity and low parity (*P*<0.001), no history of breast feeding (*P*<0.001), HRT use (*P*=0.001), premenopausal status (*P*<0.001), later age at first birth (*P*=0.02), younger age (*P*<0.001) and being Chinese (*P*<0.001). After adjusting for the well-established confounders age, BMI and ethnicity, all associations remained significant or borderline significant except for age at first birth.

Table 3 shows the results of the interaction analyses. Significant interactions with percent density were observed for BMI and oral contraceptive (OC) use (*P*=0.02 in both cases). The increasing risk with density was stronger in those with high BMI (that is, in those with high density despite high BMI), and in those with a history of OC use. Numbers of cases and controls in individual categories are available from the authors.

## Discussion

Our main findings in this Asian population are that percent breast density, as measured by the Cumulus interactive threshold, is strongly associated with risk of breast cancer. Also, percent density was a stronger associate of risk than absolute dense area, remaining substantial and significant after adjustment for other risk factors. The adjusted odds of breast cancer for those with more than 75% density was around five times that of those with density 10% or less.

We found that percent density was a stronger predictor of risk than absolute density, in agreement with some (Vachon *et al*, 2007; Torres-Mejia *et al*, 2005), but not with all studies (Stuedal *et al*, 2008; Chen *et al*, 2004). In our study, the pseudo-*R*^2^ for absolute dense area was 1% compared with 7% for percent density. However, the negative confounding with BMI was only observed for percent density. Although the regression coefficient in the conditional logistic model for percent density increased by 20% after adjustment for BMI, the coefficient for absolute dense area decreased by 4%. Others have similarly found greater confounding with BMI for percent density than for absolute dense area (Stone *et al*, 2009). The negative confounding of percent density with BMI was entirely because of a positive correlation of BMI with absolute nondense area (Haars *et al*, 2005).

We found that density was positively associated with late first birth, lack of history of breast feeding and use of HRT, but negatively associated with other breast cancer risk factors, namely age, low parity and BMI, as reported by others (Haars *et al*, 2005; Dite *et al*, 2008). We also found that risk persisted beyond 6 years after a single mammographic density reading, supporting to the hypothesis that mammographic density may be an intermediate phenotype for breast cancer (Boyd *et al*, 2005) as many of the standard risk factors are irreversible by the time of menopause.

Although percent density was higher in Chinese women in our study, we could not confirm our previous finding of heterogeneity by ethnicity of the effect of density on risk (Duffy *et al*, 2004). Although this effect was slightly stronger in non-Chinese subjects (Table 3), it did not reach statistical significance. We did, however, find significant heterogeneity of the density effect by OC use and BMI, the increased risk associated with higher density being more pronounced in those who had used OC or in those with higher BMI. The latter result may explain the long-observed negative confounding of breast density and BMI. The higher BMI in postmenopausal women is associated with both higher risk and lower percent breast density (Boyd *et al*, 1998, 2007; Stone *et al*, 2009), but higher breast density for a given BMI confers higher risk. Our results suggest that for those whose density remains high despite high BMI, there is an increase in risk more than twice as large as that expected from the combination of the two risk factors. It should be noted that the interaction tests tend to have low statistical power because of small numbers, as reflected by the wide confidence intervals on some ORs.

Our results confirm the independent effect of breast density on breast cancer risk. We found that percent breast density was a stronger risk factor than absolute dense area, but was also more confounded with BMI. Our results indicate that density is positively related to most breast cancer risk factors, with the notable exceptions of age and BMI, the effect being increased in women with high BMI or a history of OC use.

## Figures and Tables

**Table 1:** Distribution of cases and controls by potential risk factors, and results of univariate conditional logistic regression

**Factor**	**Category**	**Cases (%)**	**Controls (%)**	**OR (95% CI)**	**Significance**
Age (years)	<55	160 (33)	320 (33)	NA^a^	NA
	55–59	196 (40)	392 (40)	NA	
	60+	135 (27)	270 (27)	NA	
Ethnicity	Chinese	422 (86)	844 (86)	NA	NA
	Other	69 (14)	138 (14)	NA	
Education	None	239 (49)	577 (59)	1.00 (−)	*P*=0.001
	Primary	93 (19)	176 (18)	1.29 (0.95–1.73)	
	Secondary	107 (22)	143 (15)	1.83 (1.35–2.47)	
	A-level	37 (7)	54 (5)	1.68 (1.06–2.64)	
	Higher	15 (3)	32 (3)	1.24 (0.65–2.36)	
Percent breast density	0–10	33 (7)	117 (12)	1.00 (−)	*P*<0.001
	11–25	76 (15)	282 (29)	1.04 (0.64–1.69)	
	26–50	215 (44)	391 (40)	2.16 (1.39–3.35)	
	51–75	151 (31)	171 (17)	3.59 (2.23–5.78)	
	76–100	16 (3)	21 (2)	3.34 (1/54–7.20)	
Age at menarche	⩽14	295 (60)	514 (52)	1.00 (−)	*P*=0.003
	>14	196 (40)	468 (48)	0.71 (0.56–0.90)	
Age at menopause	<50	149 (30)	370 (38)	1.00 (−)	*P*=0.008
	⩾50	271 (55)	503 (51)	1.32 (1.03–1.68)	
	Premenopausal	71 (15)	109 (11)	1.76 (1.17–2.63)	
Parity	0	70 (14)	95 (10)	1.00 (−)	*P*=0.005
	1	30 (6)	51 (5)	0.78 (0.45–1.36)	
	2	101 (21)	167 (17)	0.82 (0.55–1.23)	
	3+	290 (59)	669 (68)	0.59 (0.41–0.83)	
Age at first birth (parous only)	<20	62 (15)	155 (17)	1.00 (−)	*P*=0.01
	20–24	146 (34)	376 (42)	0.46 (0.23–0.93)	
	25–29	139 (33)	248 (28)	0.42 (0.22–0.81)	
	30–34	54 (13)	85 (10)	0.64 (0.33–1.23)	
	35+	20 (5)	23 (3)	0.62 (0.30–1.27)	
History of breastfeeding	No	164 (39)	279 (31)	1.00 (−)	*P*=0.006
	Yes	257 (61)	608 (69)	0.69 (0.52–0.91)	
Oral contraceptive use	No	325 (66)	602 (61)	1.00 (−)	*P*=0.07
	Yes	166 (34)	380 (39)	0.81 (0.64–1.02)	
HRT (ever)	No	401 (82)	852 (87)	1.00 (−)	*P*=0.008
	Yes	90 (18)	130 (13)	1.52 (1.11–2.05)	
Family history of breast cancer	No	456 (93)	956 (97)	1.00 (−)	*P*<0.001
	Yes	35 (7)	26 (3)	2.88 (1.69–4.89)	
Body mass index^b^	<21.5	92 (19)	253 (25)	1.00 (−)	*P*=0.03
	21.5–23.89	114 (23)	244 (25)	1.21 (0.88–1.67)	
	23.9–26.69	143 (29)	248 (25)	1.51 (1.10–2.08)	
	⩾26.7	141 (29)	245 (25)	1.51 (1.10–2.07)	

Abbreviations: CI=confidence interval; HRT=hormone replacement therapy; NA=not applicable; OR=odds ratio.

a Matching variable so inference not applicable.

b By quartiles of the control group.

**Table 2:** Effect of percent density as measured by the Cumulus interactive threshold method on breast cancer risk, unadjusted, adjusted for BMI and adjusted for other significant risk factors

	**OR (95% CI)**		
**Density**	**Unadjusted**	**Adjusted for BMI**	**Multivariate adjusted^a^**
0–10	1.00 (−)	1.00 (−)	1.00 (−)
11–25	1.04 (0.65–1.68)	1.14 (0.70–1.85)	1.12 (0.68–1.84)
26–50	2.16 (1.39–3.35)	2.66 (1.69–4.20)	2.58 (1.62–4.12)
51–75	3.59 (2.23–5.78)	4.65 (2.83–7.64)	3.94 (2.36–6.57)
76–100	3.34 (1.55–7.20)	5.74 (2.54–12.95)	5.54 (2.38–12.90)

Abbreviations: BMI=body mass index; CI=confidence intervals; OR=odds ratio.

a Adjusted for BMI, age at menarche, no. of deliveries, age at first birth, OC use, HRT use and menopausal status.

**Table 3:** Analysis of potential interactions between percent density and other risk factors, after adjustment for BMI

		**OR (95% CI)**						
**Risk factor**	**Categories**	**A+B (0–10%)**	**C (11–25%)**	**D (26–50%)**	**E (51–75%)**	**F (76–100%)**	***P*-value**	***P*-value^a^**
BMI	<26.7^b^	1.00	1.32 (0.66–2.66)	2.60 (1.34–5.01)	4.13 (2.09–8.14)	3.95 (1.53–10.17)	0.3	0.02
	⩾26.7	1.00	0.88 (0.44–1.74)	2.19 (1.17–4.12)	5.24 (2.36–11.65)	9.53 (0.93–97.49)		
Age of menarche	<14	1.00	1.32 (0.62–2.77)	2.81 (1.40–5.62)	4.53 (2.13–9.63)	5.70 (1.52–21.31)	0.6	0.8
	⩾14	1.00	1.07 (0.56–2.03)	2.61 (1.44–4.71)	4.73 (2.50–8.98)	5.90 (2.13–16.32)		
Parity	Nulliparous	1.00	0.56 (0.06–5.00)	0.88 (0.11–7.16)	1.13 (0.14–9.36)	3.11 (0.20–47.90)	0.2	0.5
	Parous	1.00	1.15 (0.69–1.89)	2.74 (1.72–4.37)	4.90 (2.92–8.23)	5.54 (2.32–13.22)		
No. of deliveries	<3	1.00	0.55 (0.20–1.50)	1.33 (0.52–3.37)	2.23 (0.86–5.77)	2.28 (0.59–8.70)	0.3	0.8
	⩾3	1.00	1.36 (0.77–2.42)	3.10 (1.81–5.30)	5.29 (2.90–9.63)	8.17 (2.85–23.45)		
Age at first birth	<30	1.00	1.39 (0.78–2.48)	3.40 (1.97–5.87)	5.86 (3.23–10.65)	5.27 (1.97–14.07)	0.1	0.7
(excluding nulliparous)	⩾30	1.00	0.33 (0.08–1.33)	0.46 (0.12–1.68)	1.17 (0.30–4.58)	1.09 (0.10–11.96)		
Breastfeeding	Never	1.00	2.02 (0.79–5.19)	3.45 (1.39–8.50)	5.31 (2.05–13.70)	3.19 (0.77–13.18)	0.2	0.1
	Ever	1.00	0.83 (0.44–1.54)	2.27 (1.29–3.98)	4.69 (2.49–8.82)	6.30 (1.95–20.33)		
OC use	Never	1.00	1.11 (0.60–2.02)	3.12 (1.79–5.45)	5.60 (3.07–10.21)	6.83 (2.51–18.55)	0.1	0.02
	Ever	1.00	1.19 (0.52–2.70)	2.14 (0.98–4.62)	3.30 (1.45–7.48)	4.24 (1.09–16.51)		
HRT use	Never	1.00	1.10 (0.66–1.83)	2.34 (1.44–3.78)	4.39 (2.59–7.43)	6.13 (2.50–15.01)	0.8	0.8
	Ever	1.00	1.68 (0.30–9.32)	7.19 (1.47–35.19)	8.65 (1.74–42.93)	6.83 (0.77–60.33)		
Menopausal status	Not post menopausal	1.00	1.31 (0.11–14.71)	3.12 (0.31–30.96)	6.65 (0.66–66.33)	3.92 (0.28–53.38)	0.8	0.8
	Post menopausal	1.00	1.14 (0.69–1.87)	2.67 (1.68–4.26)	4.35 (2.60–7.28)	6.81 (2.76–16.78)		
Age at screening	<55 years	1.00	1.02 (0.29–3.49)	2.94 (0.95–9.05)	5.14 (1.61–16.39)	7.42 (1.74–31.62)	0.5	0.96
	⩾55 years	1.00	1.18 (0.69–2.01)	2.60 (1.58–4.27)	4.48 (2.56–7.82)	4.84 (1.70–13.77)		
Ethnicity	Chinese	1.00	0.92 (0.53–1.57)	2.09 (1.27–3.42)	3.80 (2.23–6.49)	4.75 (1.94–11.57)	0.4	0.7
	Non-Chinese	1.00	2.74 (0.81–9.15)	7.52 (2.38–23.75)	9.16 (2.27–36.99)	13.89 (2.07–93.51)		

Abbreviations: BMI=body mass index; CI=confidence interval; HRT=hormone replacement therapy; OC=oral contraceptive; OR=odds ratio.

a *P*-value obtained by taking the single degree of freedom interaction of two continuous variables (For example, BMI and percent density on continuous scale).

b Upper quartile boundary of BMI.
